# A guide to naming human non‐coding RNA genes

**DOI:** 10.15252/embj.2019103777

**Published:** 2020-02-24

**Authors:** Ruth L Seal, Ling‐Ling Chen, Sam Griffiths‐Jones, Todd M Lowe, Michael B Mathews, Dawn O'Reilly, Andrew J Pierce, Peter F Stadler, Igor Ulitsky, Sandra L Wolin, Elspeth A Bruford

**Affiliations:** ^1^ Department of Haematology University of Cambridge School of Clinical Medicine Cambridge UK; ^2^ European Molecular Biology Laboratory European Bioinformatics Institute Hinxton UK; ^3^ State Key Laboratory of Molecular Biology Shanghai Institute of Biochemistry and Cell Biology Chinese Academy of Science Shanghai China; ^4^ School of Biological Sciences Faculty of Biology, Medicine and Health University of Manchester Manchester UK; ^5^ Department of Biomolecular Engineering University of California Santa Cruz CA USA; ^6^ Department of Medicine Rutgers New Jersey Medical School Newark NJ USA; ^7^ Computational Biology and Integrative Genomics Lab MRC/CRUK Oxford Institute and Department of Oncology University of Oxford Oxford UK; ^8^ Translational Medicine Oncology R&D AstraZeneca Cambridge UK; ^9^ Bioinformatics Group Department of Computer Science Interdisciplinary Center for Bioinformatics University of Leipzig Leipzig Germany; ^10^ Max Planck Institute for Mathematics in the Sciences Leipzig Germany; ^11^ Institute of Theoretical Chemistry University of Vienna Vienna Austria; ^12^ Facultad de Ciencias Universidad National de Colombia Sede Bogotá Colombia; ^13^ Santa Fe Institute Santa Fe USA; ^14^ Department of Biological Regulation Weizmann Institute of Science Rehovot Israel; ^15^ RNA Biology Laboratory National Cancer Institute National Institutes of Health Frederick MD USA

**Keywords:** gene nomenclature, gene symbols, non‐coding RNA, RNA Biology

## Abstract

Research on non‐coding RNA (ncRNA) is a rapidly expanding field. Providing an official gene symbol and name to ncRNA genes brings order to otherwise potential chaos as it allows unambiguous communication about each gene. The HUGO Gene Nomenclature Committee (HGNC, http://www.genenames.org) is the only group with the authority to approve symbols for human genes. The HGNC works with specialist advisors for different classes of ncRNA to ensure that ncRNA nomenclature is accurate and informative, where possible. Here, we review each major class of ncRNA that is currently annotated in the human genome and describe how each class is assigned a standardised nomenclature.

## Graphical abstract

Working with experts in the field, the HUGO Gene Nomenclature Committee describes the standardized nomenclature assigned to all major classes of ncRNA currently annotated in the human genome.

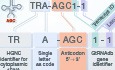

## Introduction

The HUGO Gene Nomenclature Committee (HGNC) works under the auspices of Human Genome Organisation (HUGO) and is the only worldwide authority that assigns standardised symbols and names to human genes (Braschi *et al*, 2019). A unique symbol for every gene is essential to enable unambiguous scientific communication, and approved symbols should be used ubiquitously in research papers, conference talks and posters, and biomedical databases. The HGNC endeavours to approve symbols for all classes of genes that are supported by gene annotation projects and began working on non‐coding RNA (ncRNA) nomenclature in the mid‐1980s with the approval of initial gene symbols for mitochondrial transfer RNA (tRNA) genes. Since then, we have worked closely with experts in the ncRNA field to develop symbols for many different kinds of ncRNA genes.

**Figure 1:**
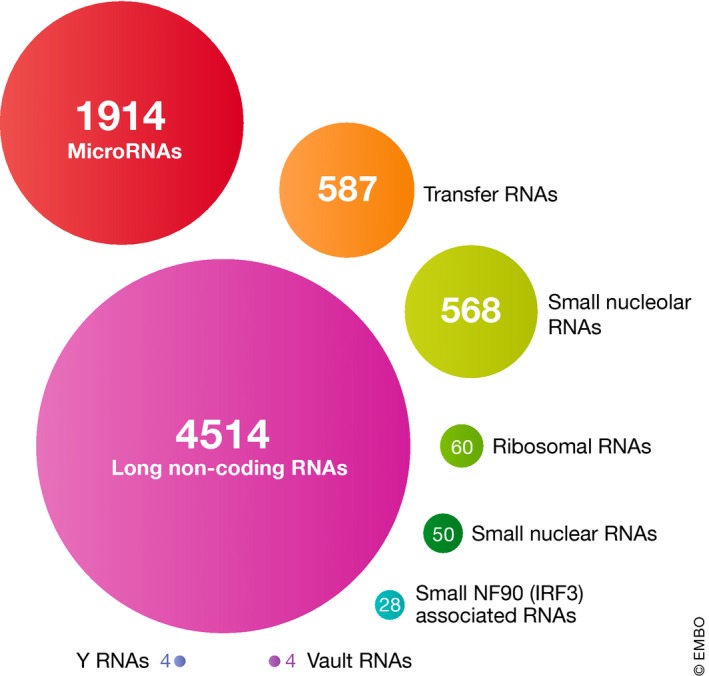
The number of HGNC gene symbols by type of ncRNA A full list of locus types, along with numbers of genes per category, can be found at our Statistics & Downloads webpage (https://www.genenames.org/download/statistics-and-files/).

The number of genes that the HGNC has named per ncRNA class is shown in Fig 1, and ranges in number from over 4,500 long ncRNA (lncRNA) genes and over 1,900 microRNA genes, to just four genes in the vault and Y RNA classes. Every gene symbol has a Symbol Report on our website, http://www.genenames.org, which displays the gene symbol, gene name, chromosomal location and also includes links to key resources such as Ensembl (Zerbino *et al*, 2018), NCBI Gene (O'Leary *et al*, 2016) and GeneCards (Stelzer *et al*, 2016). We collaborate directly with these biomedical databases and, importantly, these databases always use our gene symbols as the primary symbol for the gene. Due to the relative completeness of the HGNC ncRNA gene set, our data have been chosen as the canonical human dataset in the RNAcentral database (The RNAcentral Consortium, 2019), an RNA sequence database resource. For microRNAs, we work with the specialist resource miRBase (Kozomara *et al*, 2019), and for tRNAs, we work with the specialist resource GtRNAdb (Chan & Lowe, 2016). We display links to these resources from the relevant Symbol Report. Where available, for lncRNAs we provide specialist links to LNCipedia (Volders *et al*, 2019), a key lncRNA resource that displays HGNC gene symbols (Box 1).

**Box 1. Useful resources for non‐coding RNA genes used by the HGNC**

**Table:** 

RNA resource	Resource URL	Description
RNAcentral	https://rnacentral.org/	Centralised database of non‐coding RNA sequences collated from expert non‐coding RNA member databases, model organism databases and sequence accession databases
miRBase	http://www.mirbase.org/	Searchable database of microRNA sequences and annotations. Also hosts the miRBase registry where researchers can submit prospective new microRNAs
GtRNAdb	http://gtrnadb.ucsc.edu/	The genomic tRNA database, which contains predicted tRNA genes by the tRNAscan‐SE program for many different species
snoRNABase	https://www-snorna.biotoul.fr/	Database of human snoRNA genes; useful resource but no longer being updated
LNCipedia	https://lncipedia.org/	Database of human long non‐coding RNA sequences and manually curated lncRNA articles
Ensembl	http://www.ensembl.org/	Genome browser for vertebrate genomes that hosts the GENCODE annotation models for non‐coding RNA genes for mouse and human genes
NCBI Gene	https://www.ncbi.nlm.nih.gov/gene/	Integrated annotation and related information for many different genomes. Incudes RefSeq manual annotation of human and mouse non‐coding RNA genes

For each class of ncRNA, we host curated gene group pages on http://www.genenames.org—a list of URLs for these is shown in Table 1.

**Table 1:** The HGNC hosts gene group pages for different types of non‐coding RNA genes. These pages follow a hierarchical structure and all pages can be browsed starting at the highest‐level gene group page labelled “Non‐coding RNAs”

Gene group name	Gene group URL	Description
Non‐coding RNAs	https://www.genenames.org/data/genegroup/#!/group/475	Overview page of all non‐coding RNAs in the HGNC project. Can be used as a starting point to browse through all types of named ncRNAs
MicroRNAs	https://www.genenames.org/data/genegroup/#!/group/476	Starting page for all microRNAs, which are split into curated human families where possible. MicroRNAs not in a defined family are listed on the first page
MicroRNA host genes	https://www.genenames.org/data/genegroup/#!/group/1690	A curated list of microRNA host genes, which is split into protein coding and non‐coding subgroups
Transfer RNAs	https://www.genenames.org/data/genegroup/#!/group/478	Starting page for all transfer RNA genes, with subgroups “Mitochondrially encoded transfer RNAs” and “Cytoplasmic transfer RNAs” (this page also has the subsets “Cytoplasmic transfer RNA pseudogenes” and “Low confidence cytoplasmic transfer RNAs”)
Small nuclear RNAs	https://www.genenames.org/data/genegroup/#!/group/1819	Lists all canonical small nuclear RNA genes; variant snRNA genes are shown as a subgroup
Small nucleolar RNAs	https://www.genenames.org/data/genegroup/#!/group/844	Starting page for snoRNAs with the subgroups “Small Cajal body‐specific RNAs”, “Small nucleolar RNAs, C/D box” and “Small nucleolar RNAs, H/ACA box”
Small nucleolar RNA host genes	https://www.genenames.org/data/genegroup/#!/group/1838	A curated list of snoRNA host genes, which is split into protein coding and non‐coding subgroups
Ribosomal RNAs	https://www.genenames.org/data/genegroup/#!/group/848	Starting page for all ribosomal RNAs, split into the major subgroups “Mitochondrially encoded ribosomal RNAs” and “Cytoplasmic ribosomal RNAs”, which is further split into subtypes of rRNAs
Vault RNAs	https://www.genenames.org/data/genegroup/#!/group/852	Full list of vault RNA genes
Y RNAs	https://www.genenames.org/data/genegroup/#!/group/853	Full list of Y RNA genes
Small NF90 (ILF3) associated RNAs	https://www.genenames.org/data/genegroup/#!/group/1624	Full list of SNAR genes
Long non‐coding RNAs	https://www.genenames.org/data/genegroup/#!/group/788	Starting page for all long non‐coding RNA gene. Divided into subgroups: Long intergenic non‐protein coding RNAs, MicroRNA non‐coding host genes, Overlapping transcripts, Intronic transcripts, Antisense RNAs, Divergent transcripts, Small nucleolar RNA non‐coding host genes, Long non‐coding RNAs with non‐systematic symbols, Long non‐coding RNAs with FAM root symbol

The aim of this paper was to provide an overview for each of the main types of ncRNA that we have named, as well as a guide to how we name them. Each section has been written in collaboration with our specialist advisors for each ncRNA class: Sam Griffiths‐Jones of the University of Manchester for microRNAs, Todd Lowe of the University of California, Santa Cruz for tRNAs, Dawn O'Reilly of the University of Oxford for small nuclear RNAs (snRNAs), Peter Stadler of the University of Leipzig for small nucleolar and vault RNAs, Andrew Pierce currently at AstraZeneca, Cambridge for ribosomal RNAs (rRNAs), Sandra Wolin of the NIH for Y RNAs, Michael Mathews of Rutgers New Jersey Medical School for small NF90 (ILF3) associated RNAs, and Igor Ulitsky of the Weizmann Institute of Science and Ling‐Ling Chen of the Shaghai Institute for Biochemistry and Cell Biology for long non‐coding RNAs. We finish by outlining recommendations for the nomenclature of circular and circular intronic RNAs, which are currently lacking official nomenclature.

## MicroRNAs

MicroRNAs are transcripts of ~ 22 nucleotides that mediate the post‐transcriptional regulation of genes via direct binding to messenger RNA (mRNA) molecules. In animal cells, microRNA (miRNA) genes are usually transcribed as long primary transcripts (pri‐miRNAs), which are processed by the Drosha microprocessor complex into precursor hairpin stem‐loop sequences (pre‐miRNAs). These hairpins are exported from the nucleus to the cytoplasm, where the stem‐loop is cleaved by the Dicer enzyme to produce a ~ 22 nt duplex. One strand of the duplex associates with an Argonaute (AGO) protein and this microRNA ribonucleoprotein complex (miRNP) binds to sites in mRNAs that are complementary to the miRNA sequence, usually in the 3′ untranslated region (UTR). The Ago‐miRNP complex then recruits other proteins, which typically mediate either the degradation or translational repression of the mRNA [for a review, see (Bartel, 2018)]. Approximately 60% of all human genes produce mRNAs that can be bound by miRNAs (Friedman *et al*, 2009), so these small RNAs provide regulation for diverse biological processes across all tissue types and stages of life. As such, miRNA genes have been implicated in many human diseases including rheumatoid arthritis (Guggino *et al*, 2018), deafness (Mencía *et al*, 2009), stroke (Panagal *et al*, 2019), psoriasis (Yan *et al*, 2019), cirrhosis (Fernández‐Ramos *et al*, 2018) and several forms of cancer (Kwok *et al*, 2017).

**Figure 2:**
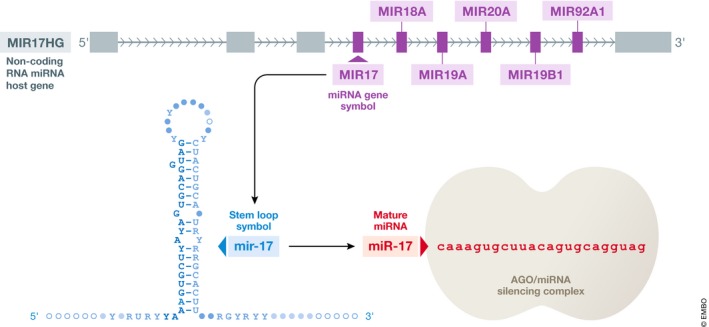
The microRNA gene *MIR17* is part of a cluster of microRNA genes that are hosted within an intron of the long non‐coding RNA gene *MIR17HG* (miR‐17‐92a‐1 cluster host gene) The symbol *MIR17* represents the gene; the symbol mir‐17 represents the miRNA precursor stem‐loop structure; and the symbol miR‐17 represents the active mature microRNA, which interacts with an AGO protein to form the AGO/miRNA silencing complex.

The name “microRNA” to reflect the small size of the active RNA molecule was agreed upon and first used by three *Caenorhabditis elegans* research groups that published in the same 2001 issue of *Science* (Lagos‐Quintana *et al*, 2001; Lau *et al*, 2001; Lee & Ambros, 2001). Once the field of miRNA research started to expand, experts came together to publish guidelines on how to name these transcripts across species (Ambros *et al*, 2003), and the miRNA Registry was founded to ensure that the same symbols were not mistakenly used by different research groups for different miRNAs (Griffiths‐Jones, 2004). The miRNA Registry evolved into the dedicated online miRNA resource miRBase, which has continued to be responsible for providing unique identifiers for miRNAs as well as acting as a database of sequences and curated publications (Kozomara *et al*, 2019). Researchers submit hairpin and mature microRNA sequences to miRBase, which are then publicly assigned new symbols after manuscript acceptance. miRBase assigns each microRNA stem‐loop sequence a symbol in the format “mir‐#” and each mature miRNA a symbol in the format “miR‐#” followed by a unique sequential number that reflects order of submission to the database. The HGNC then approves a gene symbol for human miRNA genes in the format MIR#; for example, as shown in Fig 2 and Box 2, *MIR17* represents the miRNA gene, mir‐17 represents the stem‐loop, and miR‐17 represents the mature miRNA. However, the complete extent of the miRNA gene and primary transcript is not often known, so the entity associated with an HGNC name and entry is frequently the length of the hairpin precursor miRNA, rather than the primary transcript. For genes that encode identical mature miRNAs, the same unique identifier is used followed by a hyphenated numerical suffix; e.g., *MIR1‐1* and *MIR1‐2* are distinct genomic loci that encode identical mature miRNAs. For paralogous genes that encode mature miRNAs, which differ by only one or two nucleotides, the same unique identifier is used followed by a letter suffix, e.g. *MIR10A* and *MIR10B*. The HGNC does not accept any direct requests for miRNA gene symbols, and all requests must go to miRBase first (please see http://www.mirbase.org/registry.shtml).

**Box 2. The HGNC Symbol Report for *MIR17* provides more than gene nomenclature: as highlighted here there is a link to the HGNC “MIR17 microRNA family group page”; a link out to the relevant microRNA report on miRBase; and where possible a link to the mouse ortholog at MGI and the rat ortholog at RGD**

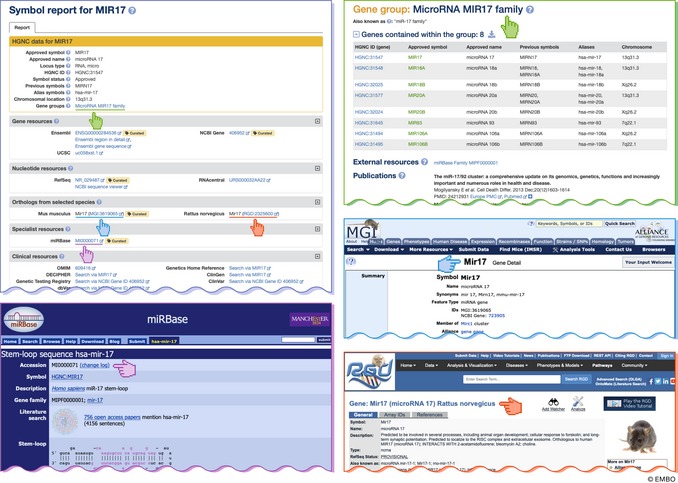

In accordance with miRBase, the HGNC provides one gene symbol per miRNA gene, even though miRNAs are sometimes processed from the same transcripts as proteins or other miRNAs, and therefore might not be considered separate genes in the canonical sense. For example, many miRNAs are hosted in the introns, or less frequently the exons, of protein coding genes or long non‐coding RNA genes (Fig 2 and Box 2). The HGNC has curated gene group pages listing these host genes (Table 1), and the naming conventions for non‐coding miRNA host genes are discussed in the long non‐coding RNA section below.

Recently, there have been a few ideas published on how to “improve” miRNA nomenclature, including correcting the identifiers of particular miRNA genes to show evolutionary relationships (e.g. Desvignes *et al*, 2015; Fromm *et al*, 2015; Budak *et al*, 2016). As nomenclature advisors, we understand the desire to perfect nomenclature systems once more information becomes available. At the same time, experience has taught us that such revised systems are often not fully adopted and may cause considerable confusion in the community. It can therefore be more appropriate to find other ways to represent relationships between genes, in order to maintain stable gene symbols. The HGNC has recently curated gene groups to show paralogous relationships between human miRNA genes, based on the family groups at miRBase and information in publications. For example, the “MicroRNA MIR1/206 family” contains the family members *MIR1‐1*,* MIR1‐2* and *MIR206*. The miRNA symbol miR‐206 has already been used in over 600 papers so it would be unhelpful to try to alter this symbol. However, the *MIR206* Symbol Report now provides a link to the curated MicroRNA MIR1/206 family gene group page, where there are also associated publications and a link through to the corresponding miRBase Family MIPF0000038 page, which lists orthologous and paralogous miRNAs in different species. Where possible, the miRNA Symbol Reports on genenames.org also display the mouse and rat miRNA orthologs, with links to the relevant gene report on the Mouse Genomic Database (http://www.informatics.jax.org/) and Rat Genome Database (https://rgd.mcw.edu/), see Box 2.

## Transfer RNAs

Transfer RNA was the first type of non‐coding RNA to be characterised over 60 years ago (Hoagland *et al*, 1958). The term “transfer” (Smith *et al*, 1959) represents the function of this RNA in transferring amino acids from the cytosol of the cell to the ribosome where the amino acids are bonded together to form a peptide according to the sequence of the mRNA being translated. Typical tRNAs vary in size from 73 to 93 nucleotides (Rich & RajBhandary, 1976) and have a distinctive cloverleaf secondary structure that folds into an L‐shaped tertiary structure (Kim *et al*, 1973). At one end of the L is the CCA acceptor site where the tRNA binds to the relevant amino acid (Hou, 2010) and at the other end is a loop that contains the three‐nucleotide anticodon which precisely pairs to the codons of mRNA (Kim *et al*, 1973). The first two nucleotides of the anticodon form Watson‐Crick base pairs with the corresponding mRNA codon, while the third nucleotide can form “wobble” pairing which allows one tRNA to recognise more than one mRNA codon. Post‐transcriptional modifications at the “wobble” position can influence binding to a particular mRNA codon (Agris *et al*, 2018).

**Figure 3:**
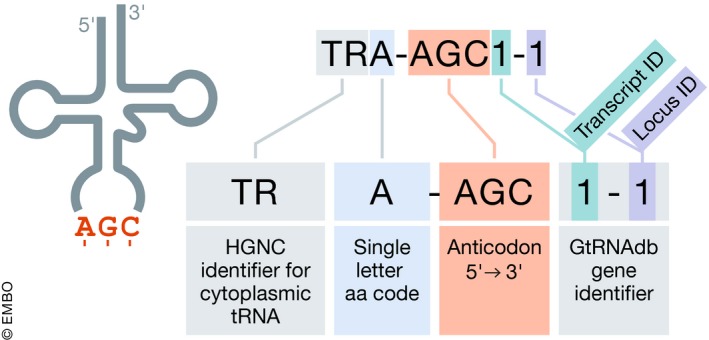
An annotated tRNA gene symbol explaining what each part of the approved gene symbol represents

Transfer RNA genes share characteristics that make it possible to predict them from genomic sequence. The Genomic tRNA Database (GtRNAdb) (Chan & Lowe, 2016) contains predicted tRNA gene sets for thousands of species across Eukaryota, Archaea and Bacteria, including a set of 429 high confidence tRNA genes for the most current human reference genome, GRCh38. tRNA gene predictions are made using the tRNAscan‐SE analysis pipeline (Lowe & Chan, 2016), which uses probabilistic tRNA primary sequence and secondary structure “covariance models” to determine the gene loci and the functional identity (i.e. tRNA isotype and anticodon) for each putative tRNA gene. The predicted tRNA genes then undergo further analysis by comparison with isotype‐specific covariance models to give confirmation of isotype classification. The GtRNAdb assigns a unique ID to each tRNA gene in the format tRNA‐[three letter amino acid code]‐[anticodon]‐[GtRNAdb gene identifier], e.g. tRNA‐Ala‐AGC‐1‐1. (Note the “GtRNAdb gene identifier” is actually made up of two numbers, the first is a “transcript ID”, the second a “locus ID”, such that multiple gene loci producing identical tRNA transcripts share the same transcript ID, but each have a different locus numbers; e.g., Ala‐AGC‐1‐1 and Ala‐AGC‐1‐2 are two different gene loci producing identical mature tRNAs, whereas Ala‐AGC‐2‐1 and Ala‐AGC‐3‐1 are genes that each produce different tRNA transcripts.) The HGNC assigns a slightly condensed but equivalent tRNA gene symbol in the format TR[one letter amino acid code]‐[anticodon][GtRNAdb gene identifier], e.g. *TRA‐AGC1‐1* (Fig 3). tRNAscan‐SE analysis also predicts tRNA pseudogenes and candidate genes that include atypical tRNA features and may not be transcribed and/or may not be capable of ribosomal translation. To reflect these different sets, the HGNC displays the gene groups “Cytosolic transfer RNAs”, “Low confidence cytosolic transfer” RNAs and “Transfer RNA pseudogenes on genenames.org” (Table 1).

The human mitochondrial genome contains 22 tRNA genes (Anderson *et al*, 1981) that encode tRNAs with both canonical and non‐canonical cloverleaf structures which enable translation within mitochondrial ribosomes in the mitochondria. While pathological mutations in cytosolic tRNA genes have not yet been discovered, mutations in mitochondrial tRNA genes cause a variety of well‐studied mitochondrial diseases such as MELAS (mitochondrial encephalomyopathy, lactic acidosis and stroke‐like episodes) and MERRF (myoclonic epilepsy with ragged red fibres) (Suzuki & Nagao, 2011; Abbott *et al*, 2014). Mitochondrial tRNA genes were named in collaboration with the MitoMap resource (Lott *et al*, 2013); gene symbols are of the format “MT‐T + one letter amino acid code”; e.g., *MT‐TA* represents the mitochondrial tRNA gene that recruits alanine. Most amino acids are decoded by just one human mitochondrial tRNA, but there are two mitochondrial leucine and serine tRNA genes—these gene symbols therefore include numbers to distinguish the individual loci: *MT‐TL1*,* MT‐TL2*,* MT‐TS1* and *MT‐TS2*.

## Small nuclear RNAs

Small nuclear RNAs are abundant transcripts of around 150 nucleotides that end in a 3′ stem loop (Matera *et al*, 2007). While the name of this RNA class is based on cellular location, each individual snRNA has a “U” identifier that stems from the historical name “U‐RNA” which was derived from early observations of their high uridine content (Hodnett & Busch, 1968). The U‐RNAs were numbered according to their apparent abundance when discovered (Chen & Moore, 2015). Some of these were subsequently found to be small nucleolar RNAs (snoRNAs) resulting in the following numbering for the snRNAs: U1, U2, U4, U5, U6, U7, U11 and U12.

Most snRNAs are involved in the splicing of introns from pre‐mRNA as part of either the major or minor spliceosome. The major spliceosome features U1, U2, U4, U5 and U6 snRNPs, plus many other non‐snRNP proteins, and performs splicing of U2‐type introns. Here, the U1 and U2 snRNPs assemble on introns and are joined by the preassembled U4/U6.U5 tri‐snRNP. This is followed by a series of rearrangements resulting in the formation of the U2/U6 catalytic core and the splicing reaction (Anokhina *et al*, 2013), and finally release of the spliced RNA and disassembly of the spliceosome. The minor spliceosome splices U12‐type introns, which make up < 0.5% of introns in the genome (Turunen *et al*, 2013). It contains the same U5 snRNA as the major spliceosome, but in contrast consists of the snRNAs U11, U12, U4atac and U6atac, which are functional analogs of the major spliceosome U1, U2, U4 and U6 snRNAs. Minor spliceosome snRNAs can fold into similar structures to their equivalent major spliceosome snRNAs, but display limited sequence similarity to them (Will & Lührmann, 2005). The term “atac” in U4atac and U6atac refers to the AT/AC splice sites found in the first U12‐type introns to be discovered (Tarn & Steitz, 1996). Instead of splicing, U7 snRNA is involved in processing the distinctive 3′ end stem loop of histone mRNA by binding to the histone downstream element and recruiting proteins, some of which shared with the spliceosome (Strub *et al*, 1984; Marz *et al*, 2007). Most snRNAs are transcribed by RNA polymerase II, with the exception of U6 and U6atac, which are transcribed by RNA polymerase III (Singh & Reddy, 1989; Younis *et al*, 2013).

All snRNA genes are named with the root symbol “RNU” for “RNA, U# small nuclear”. The GRCh38 human reference genome contains four annotated U1‐encoding loci: *RNU1‐1, RNU1‐2, RNU1‐3* and *RNU1‐4,* although individuals may have around 30 copies of tandemly repeated U1 genes (Lund & Dahlberg, 1984). The GRCh38 reference also contains a single U2 gene (*RNU2‐1*), which resides in a 6 kb region that is organised as a tandem array of 10–20 copies in many individuals (Van Arsdell & Weiner, 1984). The U7 (*RNU7‐1*), U11 (*RNU11*), U12 (*RNU12*), U4atac (*RNU4ATAC*) and U6atac (*RNU6ATAC*) snRNAs are each encoded by a single gene. There are two U4 and five U6 genes, which have numerical identifiers in the same format as the U1 genes, e.g. *RNU4‐1*,* RNU6‐2*, while the five U5 genes have letter identifiers based on the scientific literature (Sontheimer & Steitz, 1992): *RNU5A‐1*,* RNU5B‐1*,* RNU5D‐1*,* RNU5E‐1* and *RNU5F‐1*.

The human genome contains over 1,000 divergent gene copies of snRNA genes (Vazquez‐Arango & O'Reilly, 2018), most of which are presumed to be unexpressed pseudogenes. In the case of the U1 family, some of the genes present on the 1q21.1 cluster have been shown to be expressed, undergo 3′ end processing and bind U1‐specific proteins to form snRNPs *in vivo* (O'Reilly *et al*, 2013). These genes have been named with the root symbol RNVU1 for “RNA, variant U1 small nuclear”. The snRNA vU1.8, encoded by *RNVU1‐8*, has been shown to be capable of processing the 3′ end of pre‐mRNAs expressed from a subset of target genes (O'Reilly *et al*, 2013). Moreover, snRNAs encoded by *RNVU1‐3*,* RNVU1‐8* and *RNVU1‐20* are implicated in stem cell maintenance and neuromuscular disease (Vazquez‐Arango *et al*, 2016).

## SnoRNAs

Small nucleolar RNAs are transcripts of around 60–170 nucleotides that can be divided into three major classes: C/D box snoRNAs (SNORDs), H/ACA box snoRNAs (SNORAs) and small Cajal body‐specific RNAs (scaRNAs). Although some are transcribed from independent promoters, most snoRNAs are encoded within the introns of either protein coding or long non‐coding “host” genes (see Table 1 for details on accessing gene groups listing these). C/D box snoRNAs are named after their two conserved box motifs: C (sequence: RUGAUGA) and D (sequence: CUGA) (Tyc & Steitz, 1989); these snoRNAs primarily function in the nucleolus within small nucleolar ribonucleoprotein (snoRNP) complexes to direct target site‐specific 2′‐*O*‐methylation of rRNAs (Kiss‐László *et al*, 1996). H/ACA box snoRNAs share a common secondary structure and contain the AnAnnA sequence known as the “hinge” or “H” box and the trinucleotide “ACA” box (Ganot *et al*, 1997a, 1997b). H/ACA snoRNAs also function with snoRNP complexes in the nucleolus to guide modification of rRNAs, but in this case the modification is pseudouridylation of target uridines (Ganot et al, 1997a, 1997b). Small Cajal body‐specific RNAs function in the Cajal body, a nuclear organelle named after its discoverer Santiago Ramón y Cajal (Gall *et al*, 1999). ScaRNAs contain either H/ACA boxes, C/D boxes or a mixture of both types, and function as guides for the same type of RNA modifications as the nucleolar snoRNAs—guiding RNP complexes to catalyse pseudouridylation or 2′‐*O*‐methylation—but for modification of snRNAs instead of rRNAs. The major difference in sequence between scaRNAs and snoRNAs is thought to be the presence of Cajal body targeting sequences, the CAB box in H/ACA scaRNAs (Richard *et al*, 2003) or the G.U/U.G wobble stems in C/D scaRNAs (Marnef *et al*, 2014). Some snoRNAs show no sequence complementarity to either rRNAs or snRNAs, suggesting they have an alternative function to the canonical snoRNAs described above. For example, there have been recent reports of snoRNAs involved in diverse functions such as activation of enzymes, or regulation of alternative splicing and mRNA levels (Falaleeva *et al*, 2017).

When snoRNAs were first discovered, they were initially not distinguished from other snRNAs and were therefore assigned “U” numbers, e.g. U3, U8 and U13 (Tyc & Steitz, 1989), which are the identifiers still in use for snRNAs (see small nuclear RNAs section above). Once the H/ACA and C/D boxes were identified, a convention of using the root ACA# (Kiss *et al*, 2004) or HB‐I# for human H/ACA box snoRNAs and HB‐II# for C/D box snoRNAs (Cavaillé *et al*, 2000) was established, which then formed a “rival” nomenclature to the U# system that was still in use. Originally, scaRNAs were not discernible from other snoRNAs by symbol; e.g., the first identified scaRNA was referred to as U85 (Jády & Kiss, 2001) and another as ACA26 (Tycowski *et al*, 2009). In 2007, the HGNC worked with snoRNABase (Lestrade & Weber, 2006) to devise a standardised, easily recognisable nomenclature for all three types of snoRNA: SNORD# for “small nucleolar RNA, C/D box” genes; SNORA# for “small nucleolar RNA, H/ACA box” genes; and SCARNA# for “small Cajal body‐specific RNA” genes. Unfortunately, the snoRNABase resource, although still valuable, is no longer being updated. The HGNC now works with the Stadler Bioinformatics Leipzig group to assign symbols to newly identified snoRNA genes (Jorjani *et al*, 2016), and as such, the HGNC snoRNA gene group pages (Table 1) provide an up‐to‐date list of canonical human snoRNA and scaRNA genes.

A potential issue for nomenclature is that snoRNAs and scaRNAs cannot always be distinguished unambiguously without evidence of localisation. Thus, ncRNAs of these classes are by default named as SNORA# or SNORD# unless evidence for Cajal body specificity is available. Some snoRNAs are a source of miRNA‐like small RNAs; in a few cases, these small RNAs function in post‐transcriptional gene silencing like miRNAs (Scott & Ono, 2011). Interestingly, H/ACA snoRNAs are processed by Dicer, while small RNAs derived from box C/D snoRNA appear to use a different processing pathway (Langenberger *et al*, 2013). At present, HGNC does not provide a nomenclature for the small RNAs derived from snoRNA and scaRNAs.

## Ribosomal RNAs

The ribosome is responsible for the synthesis of peptides using mRNA as a template. The term “ribosome” was coined by Richard B. Roberts to provide a more user‐friendly version of “ribonucleoprotein particles of the microsome fraction” (Roberts, 1958). The ribosome, its subunits and rRNAs have all been assigned unique identifiers in Svedberg units based on their sedimentation rate in a centrifuge—the eukaryotic ribosome is referred to as the 80S ribosome and comprises a large (60S) subunit that contains 28S, 5S and 5.8S rRNA and a small (40S) subunit that contains 18S rRNA. Both subunits also contain a large number of ribosomal proteins (Khatter *et al*, 2015). The 28S rRNA forms the core of the large subunit and contains the catalytic peptidyl transferase centre (Polacek & Mankin, 2005) that forms bonds between amino acids to create peptides, meaning that the ribosome is also a ribozyme. 5S rRNA is necessary for translation (Ciganda & Williams, 2011) although its exact role is unclear, while 5.8S rRNA appears to have a role in ribosome translocation (Abou Elela & Nazar, 1997). 18S rRNA is at the core of the small subunit and binds directly to mRNA during translation initiation (Martin *et al*, 2016) and translation elongation (Tranque *et al*, 1998; Demeshkina *et al*, 2000).

**Figure 4:**
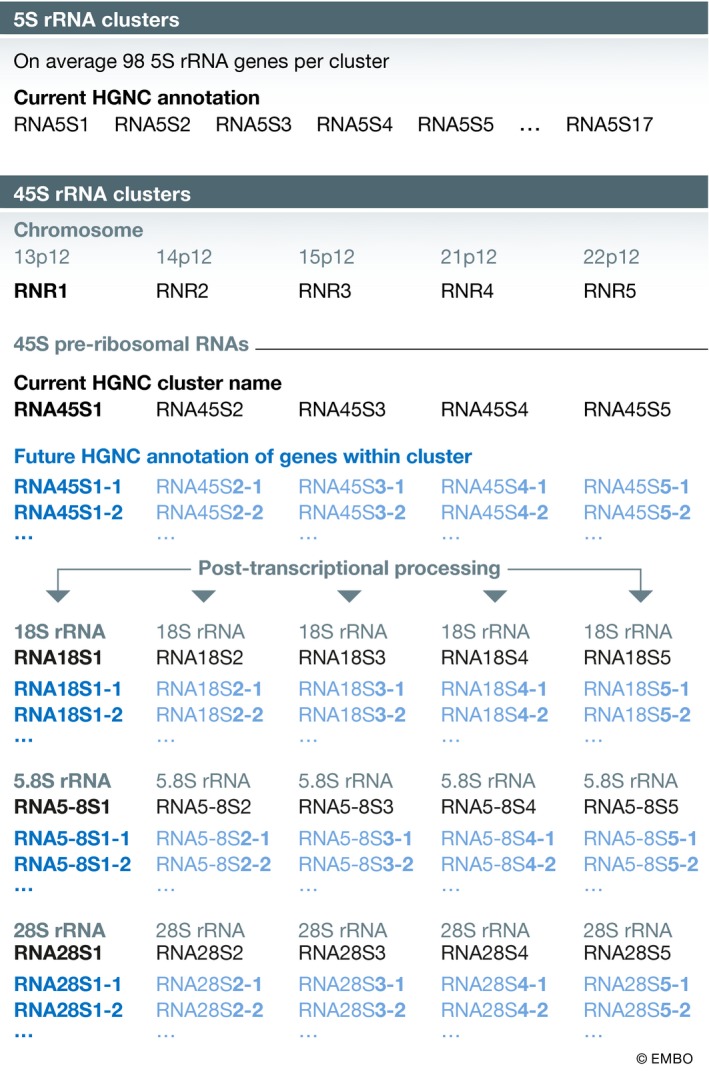
Schematic showing the two types of ribosomal RNA (rRNA) gene cluster found within the human genome The 5S cluster has a variable copy number between individuals, with 98 being the average copy number, while the current human reference genome, GRCh38, has just 17 copies. The HGNC has approved symbols for the 17 annotated copies as shown above. There are five separate 45S rRNA clusters, which are named *RNR1*‐*RNR5*. These clusters are not currently represented on GRCh38. The HGNC has approved root symbols for each 45S rRNA genes and their post‐transcriptionally processed transcripts (root symbols shown in dark blue text). The light blue symbols show the format that will be approved in the future for individual 45S rRNA genes and transcripts once the clusters are included and annotated on the human reference genome.

Cytoplasmic rRNAs are transcribed from multicopy gene clusters (Fig. 4)—the 5S rRNA cluster on chromosome 1q42.13 (Sørensen & Frederiksen, 1991) transcribed by RNA polymerase III and the 45S rRNA clusters that encode 18S, 5.8S and 28S rRNA on the p arms of the five human acrocentric chromosomes in the cytogenetically visible nucleolar organising regions (NORs) (Gonzalez & Sylvester, 2001) transcribed by RNA polymerase I. There is great variation in the number of rRNA repeats within all of these clusters both within and between different individuals. The 5S cluster is the only one in which individual genes have been annotated on the current GRCh38 human reference genome, although currently there are only 17 annotated 5S rRNA genes, while the average individual has around 98 5S genes (Stults *et al*, 2008). The HGNC has named the 17 annotated genes *RNA5S1*‐*RNA5S17*. The 45S rRNA genes have a highly repetitive nature, which has made accurate sequence assembly difficult, and as a result, no individual 45S rRNA gene is present within the NORs on the GRCh38 reference genome. The number of 45S rRNA genes per cluster differs between individuals and varies from a single gene to more than 140 repeated genes, which are usually arranged in a head‐to‐tail orientation (Stults *et al*, 2008). The HGNC has approved a gene symbol for each of the acrocentric 45S rRNA clusters: *RNR1* (13p12), *RNR2* (14p12), *RNR3* (15p12), *RNR4* (21p12) and *RNR5* (22p12; Fig 4). The 45S rRNA repeats are post‐transcriptionally processed into the rRNAs 18S, 5.8S and 28S by a series of cleavage events. The HGNC has reserved the stem symbols *RNA45S* for pre‐45S transcription units, and *RNA18S*,* RNA5‐8S* and *RNA28S* for each processed rRNA. Each acrocentric 45S rRNA cluster in turn has a set of stem symbols reserved using the same numerical identifier as the RNR cluster symbol; e.g., the symbols *RNA45S1, RNA18S1*,* RNA5‐8S1* and *RNA28S1* are stem symbols for rRNA copies from the *RNR1* acrocentric cluster. In the future, when the 45S rRNA clusters are added to the reference genome we will assign numbers to each individual gene annotated in each cluster; e.g., *RNA45S1‐1*,* RNA28S1‐1*,* RNA18S1‐1* and *RNA5‐8S1‐1* will represent the pre‐rRNA and the processed rRNAs from the first sequenced gene on *RNR1*;* RNA45S2‐3*,* RNA28S2‐3*,* RNA18S2‐3* and *RNA5‐8S2‐3* will represent the pre‐rRNA and the processed rRNAs from the third sequenced gene on *RNR2*.

While there are many 45S rRNA pseudogenes located throughout the reference genome, interestingly there are just five 45S rRNA genes that are located outside of the acrocentric 45S clusters, which appear to be transcribed and have no obvious mutations. Because these genes are outside of the 45S rRNA clusters around which the nucleolus forms and rRNA transcription takes place (reviewed in (Lam *et al*, 2005), it is unclear as to whether these genes could be transcribed into functional rRNA molecules. We have approved gene symbols for these genes, which include the letter “N” before the numerical identifier for rRNA cluster “number unspecified”; e.g., *RNA45SN1* is located at 21p11.2 and could potentially produce the rRNAs represented by the symbols *RNA18SN1, RNA28SN1* and *RNA5‐8SN1*.

Mitochondria contain their own ribosomes, known as mitoribosomes, that comprise a large subunit containing 16S rRNA and over 50 mitochondrial ribosomal proteins (MRPs) (Koc *et al*, 2001) and a small subunit containing 12S rRNA and over 35 MRPs (Cavdar Koc *et al*, 2001). While the MRPs are encoded by the nucleus, the 16S and 12S rRNAs are encoded by the mitochondrial genome (Anderson *et al*, 1981). As for the mitochondrial tRNA genes, the mitochondrial rRNA genes were named in collaboration with Mitomap (Lott *et al*, 2013)—the gene encoding 12S rRNA has the symbol *MT‐RNR1* for “mitochondrially encoded 12S rRNA” and that encoding 16S rRNA has the symbol *MT‐RNR2* for “mitochondrially encoded 16S rRNA”.

## Vault RNAs

Vault RNAs are small transcripts of roughly 100 nucleotides with a conserved panhandle‐like secondary structure that are transcribed by RNA polymerase III (Stadler *et al*, 2009). This class of ncRNA was originally discovered as part of a large ribonucleoprotein complex in rat liver that was named the vault complex due to its characteristic arches, which reminded the researchers of the arches found in the vaults of cathedrals (Kedersha & Rome, 1986). The current nomenclature for human vault RNA genes—using the root symbol “VTRNA” for “vault RNA”—was approved by the HGNC in coordination with the publication of two papers (Nandy *et al*, 2009; Stadler *et al*, 2009). The human genome contains a cluster of 3 vault genes on 5q31.3: *VTRNA1‐1*,* VTRNA1‐2*, and *VTRNA1‐3*; one *VTRNA2‐1* gene on chromosome 5q31.1; and a pseudogene, *VTRNA3‐1P* on Xp11.22. Association of vault RNAs with the vault complex depends upon binding and stabilisation by the TEP1 protein (Kickhoefer *et al*, 2001). The molecular function of the vault complex has remained elusive, while *VTRNA1‐1* has been found to function separately from the complex as a regulator of autophagy (Horos *et al*, 2019) and an inhibitor of apoptosis (Amort *et al*, 2015).

*VTRNA2‐1* has manifold functions unrelated to the vault complex, in particular in inflammation as binding partner of EIF2AK2 (also known as PKR) (Jeon *et al*, 2012; Kunkeaw *et al*, 2013). It is also a source of derived functional small RNAs (Kong *et al*, 2015). In earlier literature, it was mistakenly identified as “mirRNA‐886” and sometimes appears as “nc866”; it is, however, clearly a mammalian‐specific paralog of *VTRNA1*.

## Y RNAs

Y RNAs are small transcripts of ~ 100 nucleotides with distinctive secondary structures that are largely bound by the Ro60 protein, which is similar in structure to the TEP1 protein that binds vault RNAs (Bateman & Kickhoefer, 2003), hinting at an evolutionary relationship between these two classes of ncRNPs. These RNAs were first identified in RNP complexes that were immunoprecipitated with anti‐Ro60 antibodies from patients with systemic lupus erythematosus (Hendrick *et al*, 1981; Lerner *et al*, 1981) and were designated “Y” RNAs because they are mostly cytoplasmic, in contrast to the U class of small nuclear RNAs (Lerner *et al*, 1981). The human genome encodes 4 active Y RNA genes, which are all located on 7q36.1 and are transcribed by RNA polymerase III (Wolin & Steitz, 1983; Maraia *et al*, 1994, 1996). While the transcripts are referred to as Y1, Y3, Y4, and Y5, the equivalent approved gene symbols are *RNY1*,* RNY*3, *RNY4* and *RNY5* for “RNA, Ro60‐associated Y#”. Note there is no Y2 as this symbol was used for a short transcript that was subsequently found to be a truncated form of Y1 (Hendrick *et al*, 1981; Wolin & Steitz, 1983).

All Y RNAs contain a stem, formed by base pairing of the 5′ and 3′ ends, that includes the Ro60 binding site (Wolin & Steitz, 1984; Pruijn *et al*, 1991; Green *et al*, 1998). At the other end of this stem are one or more internal loops and stem loops that interact with other proteins to generate specialised RNPs (Sim *et al*, 2012; Chen *et al*, 2013). Y RNAs can influence the subcellular location of Ro60 (Sim *et al*, 2009, 2012) and may regulate the ability of Ro60 to bind misfolded RNAs (Stein *et al*, 2005; Fuchs *et al*, 2006; Wolin *et al*, 2013), a function supported by work in bacteria (Chen *et al*, 2007, 2013). There have also been reports of a Ro60‐independent function for mammalian Y RNAs in DNA replication (Christov *et al*, 2006; Krude *et al*, 2009), although mouse cell lines depleted of Y RNAs show no growth defects (Sim *et al*, 2009, 2012; Reed *et al*, 2013).

## SNARs

Small NF90 (ILF3)‐associated RNAs (snaRs) were first identified following immunoprecipitation of ribonucleoproteins with antibodies against NF90, an abundant protein isoform expressed from the *ILF3* gene (Parrott & Mathews, 2007). The snaR transcripts are around 117 nucleotides, show highest expression in immortalised cell lines and testis and are transcribed by RNA polymerase III (Parrott & Mathews, 2007). snaR genes are specific to great apes and evolved from an Alu repeat element followed by genomic duplication (Parrott & Mathews, 2009). Bioinformatic analysis identified nine subsets of snaRs based on sequence similarity and the HGNC agreed on the root symbol SNAR, for “small NF90 (ILF3)‐associated RNA”, followed by a unique letter for each subset and a unique number for each gene in a subset, e.g. *SNAR‐A1, SNAR‐B2 and SNAR‐C3*. SnaRs are the least well‐characterised category of small RNAs named by the HGNC and their function remains to be determined but snaR‐A transcripts bind to ribosomes, suggesting these RNAs could have a role in translational control (Parrott & Mathews, 2009).

## Long non‐coding RNAs

Before the human genome was sequenced, a small number of functional non‐coding transcripts had been identified that could not be placed into any of the categories described so far in this paper: *7SK* (encoded by *RN7SK*) (Zieve & Penman, 1976; Diribarne & Bensaude, 2009) and 7SL (encoded by three human loci: *RNA7SL1, RN7SL2* and *RN7SL3*) (Walker *et al*, 1974; Walter & Blobel, 1982) in the 1970s, and *H19* (Brannan *et al*, 1990), *BCYRN1* (BC200) (Tiedge *et al*, 1993), and *XIST* (Brown *et al*, 1991, 1992) in the early 1990s. *7SK*,* 7SL* and *BCYRN1* are all transcribed by RNA polymerase III and function via forming complexes with proteins: *7SK* is an RNA scaffold in a complex that regulates the P‐TEFb transcription factor (Diribarne & Bensaude, 2009); *7SL* is the RNA component of the signal recognition particle that targets proteins with a signal peptide to the endoplasmic reticulum (Walter & Blobel, 1982); *BCYRN1* inhibits translation via binding to eIF4A and PABP (Muddashetty *et al*, 2002; Lin *et al*, 2008). In contrast, *H19* and *XIST*, like protein coding transcripts, are transcribed by RNA polymerase II. While *XIST* has a defined molecular function in binding to and silencing the inactive X chromosome (Chow *et al*, 2005), the exact molecular function of *H19* is still not clear—it has been associated with many types of cancer and regulates several target genes by post‐transcriptional mechanisms (Gabory *et al*, 2010).

Large‐scale studies made possible following the release of the sequenced human genome in 2001 (Lander *et al*, 2001) revealed the existence of large numbers of transcripts that appear to be untranslated and, like those above, do not belong to previously defined classes of non‐coding RNAs (Kapranov *et al*, 2002; Bertone *et al*, 2004; Cheng *et al*, 2005). These were initially referred to as mRNA‐like ncRNAs because they are generally transcribed by RNA polymerase II, and are capped, spliced and polyadenylated like protein‐coding mRNAs (Erdmann *et al*, 1999; Lottin *et al*, 2002; Bompfünewerer *et al*, 2005; Széll *et al*, 2008). A 2007 study on non‐coding transcripts in human and mouse first used the term “long” to refer to transcripts of over 200 nucleotides (Kapranov *et al*, 2007), and this classification became widespread with the term “long non‐coding RNA” (or “long non‐coding RNA”) appearing in the title of 18 papers in a 2010 PubMed search, increasing to 123 papers by the year 2013 and 1,517 papers in 2018. Although the term “long non‐coding RNA” (abbreviated to lncRNA) does not truly represent a class of non‐coding RNA, it has become a useful shorthand for such transcripts of varied/unknown function and is entrenched in the scientific literature.

Functional studies have been performed for a relatively small subset of lncRNAs. The modes of action that have been described can be grouped into several different categories (see (Chen, 2016) for a comprehensive review of lncRNA by category):

1. Cis regulation of a neighbouring protein coding locus, which can be either positive or negative regulation; e.g., *TARID* binds to the promoter of, and activates, the *TCF21* gene (Arab *et al*, 2014); *PLUT* upregulates transcription of *PDX1* by affecting local 3D chromatin structure (Akerman *et al*, 2017); *FLICR* represses *FOXP3* transcription by modifying chromatin accessibility (Zemmour *et al*, 2017).
2. Trans regulation, i.e. regulating loci away from the site of transcription of the lncRNA, e.g. *NRON,* represses NFAT trafficking as part of an RNA–protein complex (Willingham *et al*, 2005); *RMST* is a transcriptional coregulator of SOX2 that influences the transcription of genes involved in neurogenesis (Ng *et al*, 2013); *THRIL* regulates *TNF* gene expression by binding to the hnRNPL protein (Li *et al*, 2014).
3. Acting as structural components, e.g. *NEAT1,* is a core RNA component of nuclear paraspeckles (Clemson *et al*, 2009); *MALAT1* has been associated with nuclear speckles (Tripathi *et al*, 2010); *FIRRE* influences nuclear architecture by binding to several different chromosomes (Hacisuleyman *et al*, 2014).
4. Acting as molecular “decoys” to titrate proteins or small RNAs away from other binding partners, e.g. the abundant lncRNA *NORAD* sequesters PUM1 and PUM2 proteins (Lee *et al*, 2016; Tichon *et al*, 2016); *GAS5* binds to the glucocorticoid receptor NR3C1 thus preventing its binding to glucocorticoid response elements in promoters (Kino *et al*, 2010). There are many papers on the binding and sequestering of microRNAs by lncRNAs (Grüll & Massé, 2019) although there is some debate over whether lncRNAs would usually be at high enough levels within cells to effectively compete for microRNAs (Ulitsky, 2018).

The HGNC provides unique gene symbols so that lncRNA genes can be discussed unambiguously. Akin to newly characterised protein coding genes, a symbol may be chosen by research groups working on a lncRNA gene if it is unique and follows the guidelines of the HGNC. The HGNC requests that all authors contact us prior to publication so that we can check any proposed new nomenclature conforms to our guidelines and, once the symbol is accepted by us, reserve it. This ensures that the approved symbol on the HGNC website (http://www.genenames.org), and on the Ensembl, NCBI Gene and LNCipedia websites, will be exactly the same as the lncRNA symbol that appears in the literature. Failure to contact the HGNC prior to publication may result in the approval of a symbol that does not match the first published symbol, e.g. *PANDAR* instead of *PANDA* (Hung *et al*, 2011), *DANCR* instead of *ANCR* (Kretz *et al*, 2012), *THORLNC* instead of *THOR* (Ye *et al*, 2018). In cases like these where we are unable to approve a symbol that appears in a publication, we contact the corresponding author of that paper to discuss an appropriate alternative. As shown by the symbols listed here, we try to approve a symbol similar to the original published symbol.

The primary rule for naming human genes is that gene symbols must be unique; i.e., the symbol does not overlap a symbol used for another human gene and ideally does not generate a high number of false‐positive hits on literature search engines. Symbols should be a short form representation of a meaningful gene name, should not be the same as a common word in the English language, should not be named after a person or place and should not include “H” for human. Although the HGNC discourages the use of punctuation in gene symbols, hyphens may sometimes be used in lncRNA gene symbols. Where known, the gene name should represent the normal function of the lncRNA gene; e.g., the full name of *NEAT1* is “nuclear paraspeckle assembly transcript 1”, and the full name of *NRON* is “non‐coding repressor of NFAT”. We appreciate that for lncRNA genes this is not always possible, and we do permit reference to expression, e.g. *BMNCR* for “bone marrow associated non‐coding RNA” (Li *et al*, 2018a), and, in some cases, disease where the association of the lncRNA with the disease is based on more than a change in expression, e.g. *PRINS* for “psoriasis associated non‐protein coding RNA induced by stress” (Sonkoly *et al*, 2005), *NBAT1* for “neuroblastoma associated transcript 1” (Pandey *et al*, 2014). Again, such cases should be discussed individually with the HGNC prior to publication.

In addition to providing a unique symbol for each named lncRNA gene, the HGNC records other alternative symbols used by different research groups, which we refer to as alias symbols. For example, the lncRNA gene *LINC00261* was first approved by the HGNC in 2012; this unique symbol first appeared in a publication in 2013 (Cao *et al*, 2013) and has since appeared in more than 25 publications. A different symbol, *ALIEN*, was used in a 2015 publication (Kurian *et al*, 2015) with no reference to the approved symbol and the symbol *DEANR1* appeared the same year (Jiang *et al*, 2015) with reference to the approved symbol in the paper but not in the title or abstract. Using or referencing the approved symbols in the title or abstract allows all papers on a particular gene to be found easily and ensures that key information will not be missed. The HGNC symbol report for *LINC00261* shows all alias symbols so that searching our database with any of the published symbols will retrieve the correct gene in our database and in other major biomedical databases such as NCBI Gene and Ensembl. Although the HGNC endeavours to record alias symbols, there is always the possibility that these may be missed and valuable data on genes lost to future interested parties if the approved symbol is not referenced anywhere else in the publication.

Where possible, we coordinate with the Mouse Genomic Nomenclature Committee to assign the equivalent symbol for orthologous human and mouse lncRNA genes. For example, the mouse ortholog of the human lncRNA gene NEAT1 has the symbol Neat1, while the mouse ortholog of human XIST has the symbol Xist (see Table 2 for further selected examples). However, it is not always straight forward to determine orthology between human and mouse lncRNA genes (Ulitsky, 2016). We require the two genes to be at a conserved syntenic location and to have detectable sequence similarity.

**Table 2:** Selected examples of lncRNA genes with equivalent approved symbols in human and mouse. For human and mouse lncRNA genes to be considered orthologous and named as such, the HGNC requires that the genes are at a conserved syntenic location and have detectable sequence similarity. Note that human gene symbols are uppercase while mouse symbols are title case, and mouse gene symbols do not contain hyphens

Human symbol	Human gene name	Mouse symbol	References
*AIRN*	antisense of IGF2R non‐protein coding RNA	*Airn*	Yotova *et al* (2008)
*DANCR*	Differentiation antagonising non‐protein coding RNA	*Dancr*	Chalei *et al* (2014)
*EGOT*	Eosinophil granule ontogeny transcript	*Egot*	Rose and Stadler (2011)
*FENDRR*	FOXF1 adjacent non‐coding developmental regulatory RNA	*Fendrr*	Grote *et al* (2013)
*FIRRE*	Firre intergenic repeating RNA element	*Firre*	Hacisuleyman *et al* (2014)
*GAS5*	Growth arrest specific 5	*Gas5*	Smith and Steitz (1998)
*HOTAIR*	HOX transcript antisense RNA	*Hotair*	Rinn *et al* (2007)
*HOTTIP*	HOXA distal transcript antisense RNA	*Hottip*	Wang *et al* (2011)
*KCNQ1OT1*	KCNQ1 opposite strand/antisense transcript 1	*Kcnq1ot1*	Gicquel *et al* (2003)
*MALAT1*	Metastasis associated lung adenocarcinoma transcript 1	*Malat1*	Ji *et al* (2003)
*MEG3*	Maternally expressed 3	*Meg3*	Miyoshi *et al* (2000)
*MIR17HG*	miR‐17‐92a‐1 cluster host gene	*Mir17hg*	Dews *et al* (2010)
*NEAT1*	Nuclear paraspeckle assembly transcript 1	*Neat1*	Clemson *et al* (2009)
*NRON*	Non‐coding repressor of NFAT	*Nron*	Willingham *et al* (2005)
*PANTR1*	POU3F3 adjacent non‐coding transcript 1	*Pantr1*	Clark and Blackshaw (2017)
*PAUPAR*	PAX6 upstream antisense RNA	*Paupar*	Vance *et al* (2014)
*PEG13*	Paternally expressed 13	*Peg13*	Court *et al* (2014)
*PVT1*	Pvt1 oncogene	*Pvt1*	Carramusa *et al* (2007)
*RN7SK*	RNA component of 7SK nuclear ribonucleoprotein	*Rn7sk*	Driscoll *et al* (1994)
*SNHG3*	Small nucleolar RNA host gene 3	*Snhg3*	Pelczar and Filipowicz (1998)
*SOX1‐OT*	SOX1 overlapping transcript	*Sox1ot*	Ahmad *et al* (2017)
*TSIX*	TSIX transcript, XIST antisense RNA	*Tsix*	Lee *et al* (1999)
*TUG1*	Taurine up‐regulated 1	*Tug1*	Young *et al* (2005)
*TUNAR*	TCL1 upstream neural differentiation‐associated RNA	*Tunar*	Ulitsky *et al* (2011)
*XIST*	X inactive specific transcript	*Xist*	Brown *et al* (1991)
*ZFAS1*	ZNFX1 antisense RNA 1	*Zfas1*	Askarian‐Amiri *et al* (2011)

As mentioned above, a relatively small fraction of the predicted total number of lncRNA genes have been cited in publications. In addition to naming published lncRNA genes, the HGNC names genes that have been annotated by the RefSeq (O'Leary *et al*, 2016) and GENCODE (Frankish *et al*, 2019) projects. These projects initially annotated lncRNA genes based on EST, cDNA and mRNA data, which provided a set of relatively high stringency, but not necessarily full‐length, transcripts. Both projects have since started to incorporate long read RNA‐Seq data, e.g. (Lagarde *et al*, 2017). Genes are annotated as lncRNAs where there is sufficient transcriptional support for a locus, but there is not sufficient evidence of protein coding potential. Assessment of protein coding potential includes assessing cross‐species conservation of a putative open reading frame (ORF), length of a putative ORF, presence/absence of encoded features such as protein domains, ribosome profiling data and evidence of peptides via mass spectrometry. Due to the constant emergence of new data, there is a certain amount of flux between the protein coding and lncRNA gene sets. Some protein coding genes have subsequently been reannotated as lncRNA genes; e.g., the gene formerly known as *C6orf48* was reannotated as a lncRNA gene and therefore renamed by the HGNC as *SNHG32*. Equally, some lncRNA genes have been reannotated as protein coding genes either due to a reassessment based on new metrics such as phyloCSF (Lin *et al*, 2011) or based on emerging evidence from new publications. For example, *LINC00083* was reannotated as protein coding gene *CLEC20A* because the ORF is conserved and exhibits a C‐type lectin domain, while *LINC01420* was reannotated as protein coding and renamed *NBDY* based on published data (D'Lima *et al*, 2017).

**Figure 5:**
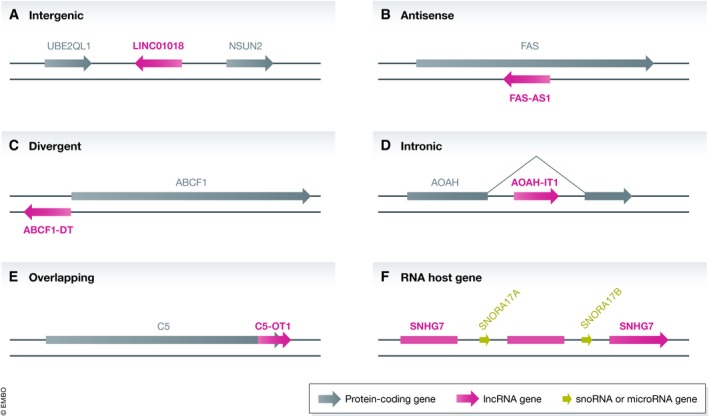
LncRNA naming schema for lncRNA genes with no published information at the time of naming LncRNAs that are intergenic with respect to protein coding genes are assigned the root symbol LINC# followed by a 5‐digit number.LncRNAs that are antisense to the genomic span of a protein coding gene are assigned the symbol format [protein coding gene symbol]‐AS#.LncRNAs that are divergent to (share a bidirectional promoter with) a protein coding gene are assigned the symbol format [protein coding gene symbol]‐DT.LncRNAs that are contained within an intron of a protein coding gene on the same strand are assigned the symbol format [protein coding gene symbol]‐IT#.LncRNAs that overlap a protein coding gene on the same strand are assigned the symbol format [protein gene coding symbol]‐OT#.LncRNAs that contain microRNA or snoRNA genes within introns or exons are named as host genes. See the main text for details on how these microRNA host genes and snoRNA host genes are named.

Feedback from conferences and research groups informed us that the lncRNA community finds genomic context with respect to protein coding genes a useful metric when considering lncRNA genes on a genomic scale. Therefore, working with the lncRNA annotation classification used by the GENCODE group, we devised a nomenclature system using the following categories (see Fig 5):

**Box 3**

1. *Intergenic lncRNA* genes are assigned the root symbol “LINC” for “long intergenic non‐protein coding RNA” followed by a unique 5‐digit number, e.g. *LINC01018* (Fig 5A). A lncRNA gene is considered intergenic (meaning between protein coding genes in this context) if it does not overlap a protein coding gene on either strand, does not share a bidirectional promoter with a protein coding gene and is not a host gene for a microRNA or snoRNA.
2. *Antisense lncRNA* genes are named using the format [protein coding gene symbol] with the suffix ‐AS and a sequential number, e.g. *FAS‐AS1* for “FAS antisense RNA 1” (Fig 5B). A lncRNA gene is considered antisense if it overlaps the genomic coordinates of a protein coding gene on the opposite strand. There does not need to be exon–exon overlap. These symbols are not intended to imply that there is a regulatory role between the protein coding and lncRNA gene. If the lncRNA is antisense to more than one protein coding gene, the symbol of the most 5′ protein coding gene will be chosen as the basis of the lncRNA gene symbol, unless there is exon–exon overlap between a more 3′ protein coding gene, which would be chosen in preference.
3. *Divergent transcripts* that are transcribed from a bidirectional promoter in the opposite direction to a protein coding gene are named using the format [protein coding gene symbol] with the suffix ‐DT, e.g. *ABCF1‐DT* for “ABCF1 divergent transcript” (Fig 5C). A lncRNA is considered divergent if it is within 300–500 nucleotides of the 5′ end of a protein coding gene on the other strand. Usually evidence of bidirectional transcription can be seen with cap analysis gene expression tags, although this is not a requirement. If a protein coding gene has multiple transcription start sites, the lncRNA will be named as a divergent transcript only if it shares the 5′ most promoter; otherwise, it will overlap the genomic span of the protein coding gene and be considered antisense.
4. *Intronic transcripts* that are transcribed entirely from within an intron of a protein coding gene on the same strand are named using the format [protein coding gene symbol] with the suffix ‐IT and a sequential number, e.g. *AOAH‐IT1* for “AOAH intronic transcript” (Fig 5D). This category accounts for a small number of our named lncRNA genes and is applied sparingly because we have found that future evidence may reveal that these loci are alternative exons or rare intron degradation intermediates of the protein coding locus.
5. *Overlapping transcripts* that overlap a protein coding gene on the same strand are named using the format [protein coding gene symbol] with the suffix ‐OT and a sequential number, e.g. *C5‐OT1* for “C5 3′ UTR overlapping transcript 1” (Fig 5E). As for the intronic transcripts above, this category is applied with caution because experience has shown us that such lncRNA genes may eventually be merged into the protein coding locus when further transcriptional evidence becomes available.
6. *Host genes* for microRNAs or snoRNAs. The small RNA may be in an exon or intron but must be on the same strand as the lncRNA (Fig 5F). LncRNA genes that host a microRNA gene are named using the format [microRNA gene symbol]HG, e.g. *MIR122HG* for “MIR122 host gene”. Where there are several microRNA genes hosted by the same lncRNA gene, the lncRNA is named after the 5′ most microRNA. If the lncRNA gene hosts a cluster, this is shown in the gene name; e.g., *MIR17HG* has the full gene name “miR‐17‐92a‐1 cluster host gene”. *MIR200CHG* hosts the microRNA genes *MIR200C* and *MIR141*; this is shown in the full gene name “MIR200C and MIR141 host gene”.

LncRNA genes that host a snoRNA gene are named using the root symbol SNHG for snoRNAs host gene followed by a unique number, e.g. *SNHG1*. This lncRNA hosts seven different snoRNA genes, so an early decision was taken to not include reference to individual snoRNA genes at the gene symbol level.

Please see the previous sections on snoRNA and microRNA genes above for more information on these small RNAs and their host genes.

In future, the HGNC will explore the possible annotation and naming of sno‐lncRNAs, a new class of transcript with a snoRNA at each end (Yin *et al*, 2012; Xing *et al*, 2017). These are processed from the introns of snoRNA host genes that host more than one snoRNA within an intron. We will also explore transcripts derived from snoRNA host genes that have a 5′ snoRNA and a poly(A) tail, which have been referred to as 5′ snoRNA capped and 3′ polyadenylated (SPAs) (Wu *et al*, 2016; Lykke‐Andersen *et al*, 2018).

The HGNC names genes and not alternative transcripts, so we assign only one name per lncRNA gene and do not provide separate symbols for non‐coding transcripts that are part of protein coding loci. Please note that the symbols in the above scheme do not mean that the lncRNA genes they represent have no function—the symbols are systematically applied where no other informative data are available at the time of naming. The HGNC will only change such symbols once future information is available where there is a consensus from groups working on these genes to do so. In some cases, our systematic symbols are already becoming well used in the literature, e.g. *LINC00473, MIR17HG, LOXL1‐AS1*. We have already named over 4,300 lncRNA genes, but we are still a long way from naming all annotated lncRNA genes; we are currently working on naming a dataset of intergenic lncRNA genes that are consistently annotated by both the GENCODE and RefSeq projects.

## Circular RNAs

Circular RNAs (circRNAs) and circular intronic RNAs (ciRNAs) are both produced during the splicing of pre‐mRNA—the major difference being that circRNAs are derived from exonic sequence, while ciRNAs are derived from intronic sequence. Currently, there are no approved symbols for circRNAs or ciRNAs; this may be a future task for the HGNC if a consensus is found in the community. CircRNAs are the result of back‐splicing of exons from pre‐mRNA, which creates a circRNA joined to itself by a 3′,5′‐phosphodiester bond (Wu *et al*, 2017; Li *et al*, 2018b). Although most of these RNAs are expressed at low levels, there are examples where the circRNA is more highly expressed than the spliced linear mRNA (Salzman *et al*, 2013). Recent studies have suggested roles for circRNAs in competitive regulation of pre‐mRNA splicing (Ashwal‐Fluss *et al*, 2014; Zhang *et al*, 2014), competitive binding to microRNAs (Hansen *et al*, 2013; Memczak *et al*, 2013), regulation of RNA polymerase II (Li *et al*, 2015) and involvement in innate immunity (Liu *et al*, 2019a). CiRNAs are derived from spliced‐out intron lariats that have escaped cleavage by the debranching enzyme. These RNAs have 2′,5′‐phosphodiester bonds between their 5′ ends and the intronic branching site creating a circular structure. Sequence analysis shows that the generation of ciRNAs is not random but depends on the presence of a consensus RNA motif containing a seven nucleotide GU‐rich element near the 5′ splice site and an 11 nucleotide C‐rich element near the intron branch point of the parent mRNA (Zhang *et al*, 2013). Knockdown of ciRNAs has been shown to reduce expression of the genes from which they are derived (Zhang *et al*, 2013).

While there is no current standardised system for naming circRNAs or ciRNAs, we suggest the following nomenclature schemes:

For circRNAs:

- circ[gene symbol]‐n where the gene symbol represents the unspliced “host” gene and n is an iterative five digit number; e.g., the first circRNA named for the host gene *PARN* would be *circPARN‐00001*

For ciRNAs:

- ci[gene symbol]‐n where the gene symbol represents the unspliced “host” gene and n is an iterative five digit number; e.g., the first ciRNA named for the host gene *PARN* would be *ciPARN‐00001*

There are currently huge numbers of circRNAs listed in public databases such as CIRCpedia (Dong *et al*, 2018), circBank (Liu et al, 2019b) and circBase (Glažar *et al*, 2014) all using different identifiers. We call on the community to come together to discuss standards creating a consensus set of circRNAs and ciRNAs that could be given standardised nomenclature in the future.

## Conclusion

In summary, the HGNC works directly with specialist advisors in the ncRNA field to ensure that appropriate and informative gene symbols are approved for ncRNA genes. We urge all ncRNA researchers to use, or at least mention, HGNC‐approved gene symbols in publications. This will ensure that ncRNA genes are correctly cited and will prevent confusion in the field. To discuss any aspect of ncRNA nomenclature, please contact the HGNC via our email address hgnc@genenames.org.

## Conflict of interest

The authors declare that they have no conflict of interest.
